# When BAT Precedes BIG‐IV in Infant Botulism: The Need for Structured Antitoxin Decision‐Pathway Reporting in Case Reports

**DOI:** 10.1002/ccr3.72956

**Published:** 2026-06-14

**Authors:** Muhammad Abdullah Awan, Hammad‐ud‐din Raza

**Affiliations:** ^1^ Wah Medical College, National University of Medical Sciences Wah Pakistan

**Keywords:** antitoxin timing, BIG‐IV, Botulinum Antitoxin Heptavalent, infant botulism, pediatric infectious disease, public health response

## Abstract

In infant botulism, use of equine‐derived botulinum antitoxin before BIG‐IV may be clinically justified when the infant‐specific product is delayed. Future case reports should document the treatment decision pathway, antitoxin timing, risk–benefit justification, guardian counseling, adverse‐event monitoring, and relapse‐prevention rationale to make such emergency decisions reproducible.

Dear Editor,

We read with great interest the case report by Gannon et al. [1] titled “Infant Botulism: A Case Study in Integrated Clinical and Public Health Response,” published in Clinical Case Reports. The authors describe a 9‐week‐old infant with constipation, feeding difficulty, progressive oropharyngeal and facial weakness, sluggish pupillary reflexes, and symmetric descending flaccid paralysis, in whom bedside nerve conduction studies supported infant botulism and fecal testing later confirmed *Clostridium botulinum* type B [1]. The report is valuable because it shows how early clinical suspicion, neurophysiologic support, intensive care, antitoxin access, and public health investigation can be integrated in a rare but high‐consequence pediatric illness.

However, one point deserves further emphasis: when Botulinum Antitoxin Heptavalent (A, B, C, D, E, F, G) [Equine] (BAT) is administered before Botulism Immune Globulin Intravenous [Human] (BIG‐IV) in an infant, the decision pathway should be reported in a structured manner. In this case, BAT was administered on Day 4 of hospitalization because it was the only readily available antidote in Ireland, while BIG‐IV was requested from the Infant Botulism Treatment and Prevention Program in California and administered later on Day 7 [1]. This sequence is understandable, but it raises important clinical questions for readers in countries where BIG‐IV is not immediately accessible.

Future reports should clearly state the exact intervals from neurological symptom onset, public health notification, bedside electrodiagnostic support, BAT decision‐making, BIG‐IV request, product arrival, and administration. Such details would help distinguish delay caused by diagnostic uncertainty from delay caused by product access. For rare diseases requiring international procurement, these intervals are not administrative details; they determine whether other hospitals can reproduce a safe emergency response.

The risk–benefit discussion around BAT also warrants explicit reporting. Gannon et al. [1] appropriately note that equine‐derived antitoxin is not routinely used in infants because of concerns such as anaphylaxis, serum sickness, and sensitivity to equine proteins, yet the infant in their case experienced no adverse effects from either BAT or BIG‐IV. Future case reports should specify how guardian counseling was performed, whether hypersensitivity precautions were used, what monitoring occurred during and after infusion, and how clinicians planned surveillance for delayed serum sickness. This would be especially useful when BAT is used as a bridge while awaiting BIG‐IV.

The rationale for dual‐antitoxin sequencing should also be made more operational. The authors explain that BAT was given first for immediate availability, whereas BIG‐IV was later used because infant intestinal colonization may persist for weeks to months and BIG‐IV provides longer protection [1]. Future reports should present this as a decision algorithm: when to give BAT, when BIG‐IV remains indicated after BAT, and which clinical or laboratory markers should prompt relapse monitoring.

Finally, the public health investigation was impressively broad, including assessment of honey, gripe water, probiotic, soother dip, well water, farm exposure, construction‐related dust, and household environmental risks [1]. Future reports could strengthen reproducibility by presenting exposures in a simple matrix: tested and negative, unavailable for testing, plausible but unproven, and excluded. This would allow clinicians and public health teams to separate source investigation from source attribution.

Overall, Gannon et al. [1] provide an important case showing that infant botulism management may require both clinical urgency and public health coordination. The broader lesson is that antitoxin availability, not only antitoxin efficacy, shapes real‐world care. Future infant botulism reports should therefore describe the full antitoxin decision pathway, especially when BAT precedes BIG‐IV.

## 1 Conclusion

This case successfully demonstrates integrated clinical and public health management of infant botulism. However, when BAT is used before BIG‐IV, future reports should include exact decision timelines, access barriers, consultation pathways, guardian counseling, hypersensitivity and serum‐sickness monitoring, dual‐antitoxin rationale, relapse surveillance, and structured exposure‐source reporting. These details would transform a successful case response into a reproducible emergency framework for settings without immediate BIG‐IV access.

## Author Contributions

**Muhammad Abdullah Awan:** conceptualization, data curation, investigation, formal analysis, project administration, writing – original draft, writing – review and editing. **Hammad‐ud‐din Raza:** data curation, investigation, writing – original draft, writing – review and editing, formal analysis.

## Funding

The authors have nothing to report.

## Ethics Statement

Ethics approval was not required for this Letter to the Editor because it discusses a previously published case report and does not include new patient data.

## Consent

Patient consent was not required for this Letter to the Editor because no new identifiable patient information is presented.

## Conflicts of Interest

The authors declare no conflicts of interest.

## Data Availability

Data sharing not applicable to this article as no datasets were generated or analysed during the current study.
